# Connecting families: a qualitative study examining the experiences of parenting young children under financial strain in Ontario, Canada

**DOI:** 10.1186/s12889-024-18463-4

**Published:** 2024-03-28

**Authors:** Mary I. Martin, Dane Mauer-Vakil, Cornelia M. Borkhoff, Patricia C. Parkin, Imaan Bayoumi

**Affiliations:** 1 Centre for Studies in Primary Care, Queen’s University, 220 Bagot St, K7L 5E9 Kingston, ON P.O. Bag 8888, Canada; 2 School of Public Health Sciences, University of Waterloo, Waterloo, ON Canada; 3 Pediatric Outcomes Research Team (PORT), Division of Pediatric Medicine and SickKids Research Institute, Hospital for Sick Children, Toronto, ON Canada; 4 Institute of Health Policy, Management and Evaluation, University of Toronto, Toronto, ON Canada; 5 Department of Pediatrics, Temetry Faculty of Medicine, University of Toronto, Toronto, ON Canada

**Keywords:** Financial strain, Qualitative research, Families, Young children

## Abstract

**Background:**

There is little research investigating the subjective experiences of parenting young children while living in poverty and experiencing financial strain using qualitative methodologies. Therefore, the objective of this study was to employ a qualitative approach to provide a nuanced and balanced view on the topic of parenting young children under financial strain in the Canadian context.

**Methods:**

We conducted a qualitative study using semi-structured interviews between July and August 2021 in Kingston, Ontario, Canada. Sixteen participants aged 20–39 self-identified as living under financial strain while parenting a child aged 2–5 years. A qualitative inductive thematic analysis was undertaken with a focus on describing the contents of the data.

**Results:**

Four major themes emerged from the data: experience of being a parent, impact of financial strain on the family unit, impact of financial strain on the children, and impact of financial strain on the parent. Numerous deleterious physical, mental, and material impacts on the family unit and parent were identified, however parent-perceived impacts of financial strain on their children were minimal. Parents described striking levels of resourcefulness and resiliency in providing the necessities for their families, absorbing the most significant impacts of financial strain through the phenomenon of self-sacrifice.

**Conclusion:**

The impacts of financial strain on families with young children are far reaching. Further research into the impacts of self-sacrifice on parents experiencing financial strain are needed to better understand this issue, and to inform social programming and resources that could help alleviate the deleterious impacts of poverty on parent mental, social, and physical health.

**Supplementary Information:**

The online version contains supplementary material available at 10.1186/s12889-024-18463-4.

## Background

Poverty and material hardship have profound detrimental impacts on health, contributing to significant health disparities in domains such as parenting stress and mental health issues, as well as children’s psychosocial development [1, 2]. Given that such financial difficulties can negatively contribute to children’s behavioural issues, mental disorders, and physical health problems, it represents a significant public health concern [3, 4]. Socioeconomic health disparities are estimated to cost the Canadian health system $6.2 billion annually [5], while investments in early childhood for structurally disadvantaged children such as home visiting programs and early childhood education have been linked to improved health, social, education and economic outcomes and a return on investment of 7–10% annually [6].

Financial strain refers to a specific stress arising from economic difficulties, including financial insecurity, poor credit, and high debt [7]. While poverty is specifically linked to income level, financial strain is not; it is a subjective psychological experience [8]. Financial strain is correlated with parenting satisfaction and self-efficacy among parents of adolescents and children [9, 10]. It is associated with negative impacts on adult mental health, including increased risk for depression and anxiety symptoms and diagnoses [11, 12]. Resource scarcity affects adult psychological functioning and cognitive processes, which can manifest through negative emotions such as stress and worry [13–16]. In a recent qualitative study, Marcil and colleagues [7] found that for women parenting young children, financial strain resulted in forced trade-offs, compromised parental practices, and self-blame, contributing to increased mental health problems.

Financial concerns also influence parents’ attitudes and behaviours associated with parenting, impacting their capacities to interact positively with their families [17]. Negative associations between economic strain, parental self-efficacy, and parental satisfaction have been documented [9, 18, 19]. Financial difficulties can affect the relationship quality between parents and children [20], and parental mental health, including depression and anxiety, can contribute to a deterioration of positive parenting practices [21–24].

While there are extensive bodies of work investigating the impact of poverty and financial strain on the lives of parents, adolescents, children, and families, there is a limited literature examining the subjective experiences (both positive and negative) of parenting young children under such conditions using qualitative methodologies. Therefore, the objective of this study was to employ a qualitative approach to provide a nuanced and balanced view on the topic of parenting young children under financial strain in the Canadian context.

## Methods

### Study design & setting

This qualitative study was conducted in Kingston, Ontario, Canada, a city with a population of 136,685, between July and August 2021. We used a phenomenological approach to provide an in-depth perspective on a common experience and behaviours in a specific population [25]. The study was reviewed for ethical compliance by the Queen’s University Health Sciences and Affiliated Teaching Hospitals Research Ethics Board (file no. 6,024,832).

### Participants & recruitment

A convenience sample was drawn from participants of the Connecting Families pilot study, a project investigating the role of a community support navigator to assist families with young children with accessing local social services [trial registration NCT05091957 at clinicaltrials.gov]. Eligible individuals were parenting a child 2–5 years of age, lived in Kingston, Ontario, and answered yes to: “Do you ever have difficulty making ends meet at the end of the month?” We focused on this preschool age group because there is evidence of mental health disparities by school entry, and that reduced duration of poverty exposure is associated with improved mental health, even by age 5 years [26]. A minimum age of 2 years was chosen, as the primary outcome in the corresponding quantitative study is not valid for children younger than 2 years. Participants were actively recruited by one researcher (MM) from a family medicine clinic, specialty children’s clinic, several early years centres, as well as a social assistance office. Recruitment notices were also posted in libraries and disseminated via Facebook. Participants who had completed participation in the Connecting Families study, or who were in active follow-up were eligible for inclusion (*n* = 25); those who were lost to follow up were ineligible (*n* = 14). Eligible individuals were phoned or emailed by one researcher (DMV) and invited to participate in an interview. Written informed consent was obtained from all participants. Following the interview, all participants received $50.00 as an honorarium.

### Data collection

Of the eligible participants, two declined, seven were unreachable, and sixteen completed an interview. One researcher (DMV) conducted 30–60 min individual semi-structured interviews by telephone (to comply with COVID-19 public health restrictions). Interview questions, developed by the research team who have experience in qualitative inquiry, included participants’ general experiences as parents as well as various impacts of financial strain (Appendix A).

### Data analysis

Inductive thematic analysis was completed using open coding in NVivo 12 (QSR International) [27, 28]. Research team members (IB, MM, DMV) independently read and coded two transcripts to develop a preliminary codebook, which was refined and used to code the remaining transcripts. All transcripts were double-coded. Preliminary codes were reviewed to identify emerging themes, which were then progressively combined through consensus to the final four major themes. Exemplary quotes were selected to illustrate key concepts. Discussions around researcher assumptions/bias were held throughout the analytic process to address reflexivity, which may have otherwise impacted data analysis and interpretation of the results [29]. Discussions involved challenging assumptions made about the data that were informed through prior knowledge of the local area, services, and communities familiar to the researchers. We took care to interpret results based only on the data gathered from participants in the current study rather than allowing prior work with similar populations impact the lens through which we viewed the data.

## Results

Participant demographics are summarized in Table 1.

**Table 1:** Participant Demographics

Characteristics	No. of participants *n* = 16
**Gender Identity**	
Male Female	1 15
**Age (years)**	
20–24 25–29 30–34 35–39	4 2 8 2
**Marital Status**	
Married Common Law Single	5 4 6
**Ethnicity**	
European Indigenous	12 3
Other	1
**Household Unit**	
Single mother + child(ren) Two parents + child(ren) Multi-generational Single mother + child(ren) + non-related roommate	4 10 1 1
**Number of children**	
1 2 3 4 5	3 6 4 2 1
**Education**	
Some high school High school diploma College diploma University degree	2 7 5 2
**Employment– participant**	
Unemployed Employed part-time Employed full-time Full-time student	11 2 2 1
**Employment– spouse**	
No spouse	6
Unemployed	3
Employed full-time	7
Household Income last year*	
<$10 000 $10 000-$19 999 $20 000-$29 999 $30 000-$39 999 $40 000-$49 999 $50 000-$59 999 $60 000-$79 999	4 5 4 1 1 0 1

*income recorded at date of final survey

ϮSpouse– married or common law partner

Four major themes were identified: experience of being a parent, impact of financial strain on the family unit, impact of financial strain on children, and impact of financial strain on the parent.

### Experience of being a parent

When asked to describe their experience as a parent, many participants expressed positive feelings, noting that they enjoyed being a parent.

*“If you really love doing it then it’s one of the best feelings in the world.”* - P08.

The best parts of parenthood were described as experiencing the growth and development of their children and the feeling of love and connection to their family.

“*…watching them grow into their own. Like I have four kids with four completely different personalities, and it’s just kind of cool to see how different they are in some things, but how similar they are in others, if that makes sense. Yeah it’s just cool to watch and to know that I had a fair size part in some of the things.”*– P16.

They also described their experience of parenthood as a dichotomy of good and bad,

*“It’s extremely difficult but the most rewarding thing.”* - P07.

and described challenges of parenthood including behavioural issues, as well as the financial strain associated with raising a family.

“*So I think it was just like, knowing that we were going to fall short. And I’m not saying short as in like $50. I’m saying short as in like a few hundred dollars each month and worrying about how we were going to feed our children or pay our bills. It was mentally exhausting*.” -P02.

### Impact of Financial strain on the Family Unit

The most common factor contributing to financial strain was the rising cost of living, including necessities such as rent and food, which was further compounded by the family’s low income. This was especially true among those who were or had been receiving social assistance.

*“I’m on [social assistance] and I don’t get much. I only get around $250 after they pay my rent. And I struggle with buying groceries and paying bills.”* -P06.

Nearly half of participants had or were currently receiving financial support through social assistance programs, three quarters had accessed resources related to food insecurity (such as food banks, meal hampers or hot meal programs), and half had accessed help from informal sources such as receiving food or money from a friend or family member.

*“We struggle with getting groceries so I end up having to resort to the Food Bank or calling family and friends to receive money or like borrow money from them to get through the rest of the month. I go without my cell phone for months at a time because I can’t afford to pay my bill.” -*P09.

The most common impact of financial strain was not enough money to pay for necessities. This often led to the parents needing to prioritize the ‘most important’ necessities like food and rent over less critical bills including utilities, phone, and internet.

*“I can’t have my kids go without eating, right. So if I had to buy groceries and put food on the table or pay a bill, it was like, yes I know both are very important but in the same sense my kids need to eat, right?”* -P02.

Financial strain also led to conflict in the family, especially between spouses/parenting partners.

*“It caused a lot of tension, you know, I would blame him, he would blame me like why we couldn’t provide and stuff like that. And we ended up separating. We were young, obviously, but we were together ten years and like all the struggle and homelessness and stuff made us separate.”* -P15.

Despite financial strain touching many aspects of the family’s life, many participants describe the phenomenon of community building that resulted from experiencing financial hardship alongside neighbours, friends, and family. They described sharing resources like food, childcare, clothing and toys, and even lending money when possible.

*“We lived in a pretty good neighbourhood… like the [area] isn’t known to be the greatest, but we had quite some amazing neighbours that would go through their cupboards and give us what they, whatever they could, which was great… I could never be more thankful for the community that we had.”* -P02.

Nearly two-thirds of participants felt that their struggle had led to a strengthening of their familial relationships, bringing the family closer and encouraging open and effective communication.

*“It just made us stronger, you know. Like we’ve gone through a lot of stuff in our 12 years together and every day it just makes us stronger as a couple and as a family.”* -P02.

### Impact of Financial strain on children

Many parents felt that their children’s lives weren’t significantly impacted by their financial struggles, especially if the children were young and unaware of the impacts on their lives.

*“I wouldn’t say my financial situation really impacts them a lot though because obviously they’re still young. They don’t know my financial situation.*” -P15.

Though most parents noted that their child never went without their needs, living with financial strain often meant that children couldn’t have all their wants, like toys, electronics, or new clothes.

*“I feel that my child misses out on new toys. He gets sad that like his friends get new things all the time and he can’t.”* -P06.

When reflecting on their children’s social connections, many parents noted that their children always had friends and that their social lives were not impacted by their financial troubles. However, some noted they missed out on experiences with friends if there was an associated cost, like day trips or birthday parties.

*“Sometimes they’ve missed a birthday party or something because I don’t have the money to buy a gift, so I won’t send them to the birthday. So that probably would affect their social life as far as friends.”* -P15.

Similarly, participants noted that their children often missed out on other experiences like class trips, extracurricular activities, and family vacations when money was tight.

*“I mean I’m sure there [are things they miss]. Vacations or, you know, doing something fun for the summer. It was easier when you had one or two kids. Whereas four, it’s a lot more costly.“* -P16.

Parents noted some positive impacts on their children, however, such as learning the value of money, the importance of prioritizing needs over wants.

*“And I thought, teaching them the value of a dollar was probably one of the big ones. That just because we’re walking by a store or are in a store and you want something, doesn’t mean that you’re necessarily going to get it. Or my oldest doing chores to earn money to be able to buy that particular thing that he wants.”* -P02.

### Impact of Financial strain on parents

Nearly all parents described negative emotions associated with financial strain, including worry and stress, feeling like a failure or that they are to blame for their family’s struggles, and feeling guilt and embarrassment when having to ask for help.

*“So I’d plan out the meals, making sure they had their meals and sometimes I wouldn’t. But I would feel bad because why can’t I simply provide for my kids, you know. It makes me feel like a failure somewhat, but then again I’m my own worst critic. I feel so bad, as a failure, like why am I not doing something to make it better.” -*P04.

Half of the participants described either developing new mental health concerns or a worsening of their existing symptoms (especially anxiety and depression).

*“I’ll stay up and think, ‘how am I going to do this, how am I going to pay for that, how am I going to make it through until this date.’ It definitely gives you serious anxiety when you have people that depend on you and you need to support with very little income.” -P15*.

Nearly all parents believed their social lives had been impacted by their financial situation, with just over half admitting they have no social life.

*“If [my friends] are going out to the patios downtown, I typically won’t go because I have to budget for my whole month and for my kids…So that means I miss out on hanging out with my friends because I can’t. My kids come first.” -P15*.

Despite the material, emotional, physical, and social impacts of financial strain, many parents did their best to protect their children’s wellbeing, often sacrificing their own needs, mental and physical health. This self-sacrifice manifested as delaying the purchase of clothing, declining social activities, and even skipping meals. They viewed ensuring that their children had food as their highest priority and described occasions where they compromised their own nutritional needs to ensure their children had enough.

*“I don’t think he ever knew that we didn’t have money…or that we live cheque to cheque. I think he has no idea because I always put him first. He always has nice clothes. Nothing’s ever torn or stained. And if I have to, I will go without a meal or two…I’ve skipped meals numerous times.”* -P03.

Further, parents described a striking level of resourcefulness to ensure their children’s needs were met. This included learning new skills, problem solving, as well as a high levels of resiliency.

*“My kids have never gone without anything they needed because I always make it happen. If I can’t buy them a brand-new pair of shoes, I’ll go to a Thrift Store and find a decent pair of used shoes.”* -P15.

Many participants also described learning to effectively manage the family’s finances despite their low income.

*“I budget very good now. I know winter is coming, so I’m going to save money to buy them snow suits and boots and all this stuff, right. I knew school was coming so I budgeted to buy. I worked more to buy them new clothes for school and new shoes.”* -P12.

## Discussion

We explored the experiences of 16 individuals raising young children while experiencing financial strain. Four major themes were identified: experience of being a parent, impact of financial strain on the family unit, impact of financial strain on children and impact of financial strain on the parent. Numerous deleterious physical, mental, and material impacts on the family unit and parent were identified, however parent-perceived impacts of financial strain on their children were minimal. Parents described striking levels of resourcefulness and resiliency in providing the necessities for their families, most often at the expense of their own wellbeing.

Financial strain was a significant challenge in parenthood. The most common impact of financial strain on the family unit was the inability pay for necessities, with parents prioritizing essential needs like rent and food over all else. Despite careful planning, three-quarters of participants had accessed local food banks or meal programs to alleviate food insecurity. In 2021, food insecurity, the experience of inadequate access to food due to financial constraints [30], was experienced by 5.8 million Canadians, including 1.4 million children [30]. It has long been associated with markers of social and economic disadvantage, with the lowest-income households, and those reliant on public income supports at highest risk [30]. As most of our study population reported household income at or below the Market Basket Measure, Canada’s official poverty line [31], and nearly half had received social income support, their experiences with food insecurity are consistent with these findings. Further, food insecurity has been previously associated with increased risk of depression and anxiety symptoms and diagnoses [32, 33], which was common among participants.

Family conflict, especially between spouses, was another reported impact of financial strain. According to the Family Stress Model, economic strain and pressure disrupts family relationships through a deterioration of parental mental health, leading to problems in the interparental or spousal relationship, disrupted parenting and abnormal child psychosocial development [3, 34–37]. Previous literature has also reported that financial problems are associated with decreased marital satisfaction [38, 39], marital instability [40], increased negative communication [41], as well as hostile, contemptuous, and angry behaviours [42]. Consistent with the FSM model and this past research [43], participants in our study described arguments, miscommunication, and breakdown of relationships during times of financial hardship.

Despite these conflicts, participants perceived a strengthening of familial relationships. This is consistent with family resilience, which has been defined as the ability of a family to respond positively to an adverse situation and emerge feeling even more strengthened, resourceful, and confident than before [44]. Recollections of resiliency, creativity, and resourcefulness in the face of financial strain among participants in our study were common, especially when it came to providing food and other resources for their children. Literature on family resilience is extensive, with much focusing on understanding protective factors that contribute to a family’s resiliency [45, 46]. Protective factors, such as a positive outlook, communication, family cohesion, and self-efficacy are associated with higher levels of family resilience and were consistent among our study population. Additionally, participants described a strong sense of community and support among friends and family who were also experiencing poverty, which is supported by research noting that social support [47, 48] and involvement in the community [49] are protective factors contributing to family resilience.

The negative impact of financial strain on parents’ wellbeing was far-reaching and significant. Nearly all participants described negative emotions related to financial strain, including stress, feelings of failure, guilt, and embarrassment. The literature supports these findings, demonstrating that resource scarcity affects adults’ psychological functioning and cognitive processes [13–16], which can manifest through negative emotion such as stress and worry. For women parenting young children, financial strain has been observed to result in forced trade-offs, compromised parental practices, and self-blame, which contribute to increased mental health issues [7]. Resource scarcity has also been found to contribute to depression [11], especially in mothers [7, 12]. This is consistent with our findings, as half of our primarily female participants described developing new or worsening of existing mental health issues and symptoms during periods of financial hardship.

Another major impact on participants was self-sacrifice, which we define as parents taking on a larger share of material hardship to moderate the impacts on their children. The same phenomenon has been reported in a study which found that mothers living with financial hardship routinely reduced their own food intake to ensure their children were fed and bills were paid [50]. This study also highlighted maternal social isolation due to financial hardship, which was nearly universal among our participants. These descriptions of self-sacrifice suggest that the impact of financial strain on parents is even greater than their household income may indicate. Further research into the implications of this phenomenon on the deleterious impact on parental mental and physical health are urgently needed.

Parents perceived little effect of financial strain on their children’s lives. In contrast, there is a large body of empiric evidence of the negative impacts of childhood poverty on several dimensions of health, development, and well-being [51–55]. Further, some participants believed their children were not aware of their family’s financial strain. While studies on this topic are scarce, existing literature reports the contrary; school-aged children in low-income households are aware of their family’s financial situation and have fears of being marginalized as a result [56, 57], and there is evidence that children as young as 3 years have the cognitive capacity to recognize socioeconomic disadvantage and label themselves accordingly [58]. This suggests that an inconsistency exists between the lived experience of being a child in a low-income household and their parents’ perception of their experience. In our study when parents were asked about their children’s social lives, despite initially stating their children’s social lives were not impacted, many of the same participants also endorsed that they often missed out on activities and experiences with friends (which interestingly was also a self-identified negative impact for parent participants themselves). Existing literature on the topic reveals that children living in poverty are acutely aware of being left out of opportunities and activities, and report feelings of social insecurity and anxiety around ‘fitting in’ [56, 59, 60]. This disconnect may be in part due to parental bias towards perceiving resilience in their children, or perhaps a moderating effect due to other families in their social networks experiencing similar financial constraints and associated impacts. Additionally, it may feel incongruous to parents that their young children are negatively affected by their financial situation, given the substantial self-sacrifice reported by participants. Further research should examine this phenomenon to better understand this inconsistency.

### Limitations

The transferability of findings to other contexts may be limited due to the convenience sampling approach used for recruitment. Further, generalizability may be limited in this qualitative study due to the small sample size and concentrated geographic location of our study population. The sample population was primarily female and self-identified as European, so experiences may differ for those from other cultural groups. This gender imbalance was not surprising given the high proportion of participants recruited at early-years centres during daytime hours, and the fact that significantly higher proportions of females provide caregiving to their young children compared to males [61]. Nonetheless, this may bias our results, as women are more likely to report negative impacts of caregiving, such as feeling tired, worried, or anxious [61], and single mothers are at increased risk of negative psychological wellbeing [10, 50]. Therefore, our results may not be representative of male parent experiences.

In addition, since all participants lived in the city of Kingston, the experiences of rural dwelling families may also differ. The voluntary nature of recruitment may have also contributed to selection bias. It is possible that the participants were more socially connected, resilient and resourceful, than the general population of families struggling with financial strain. Finally, participant recollections of past experiences may be subject to recall bias.

## Conclusion

The impacts of financial strain on families with young children are far reaching. Though some evidence for protective factors that contribute to family resilience were found among our study population, numerous deleterious physical, mental, and material impacts on the parent and family unit were identified. We observed the most significant impacts on the parents themselves due to the phenomenon of self-sacrifice. Further research into the impacts of self-sacrifice on parents experiencing financial strain are needed to better understand this issue, and to inform social programming and resources that could help alleviate the deleterious impacts of poverty on parental mental, social, and physical health.

### Electronic supplementary material

Below is the link to the electronic supplementary material.

Supplementary Material 1

## Data Availability

Data sharing not applicable to this article as no datasets were generated or analysed during the current study. Please contact the corresponding author, Dr. Imaan Bayoumi, for more information.
