# Supplementary material for: Connecting families: a qualitative study examining the experiences of parenting young children under financial strain in Ontario, Canada
Source: BMC Public Health. 2024 Mar 28;24:913. doi: 10.1186/s12889-024-18463-4 (PMC10976761; doi:10.1186/s12889-024-18463-4)
Supplement: Supplementary file 1 — Supplementary Material 1 [file 12889_2024_18463_MOESM1_ESM.docx]

**Appendix A: Interview Script**

**Part 1: Introductions**

1. Please state:
   1. The gender you identify with
   2. Your age
   3. How would you identify your race/ ethnicity? Please select all that apply:
      1. Indigenous
      2. White/ European
      3. Black/ African/ Caribbean
      4. Person of colour
      5. Other – please describe.
   4. What’s your marital status?
   5. Tell me about your educational experience (highest level achieved)
   6. Tell me about your household - who do you live with?
      1. How many children do you have? What are their current ages?
      2. What is their living situation (i.e.. Live with you full time, live in two households, live with someone else (who?))
      3. Do you have a spouse/parenting partner? If so, do they live with you?
      4. Do any other adults live in your home (ex. Extended family like parents, grandparents, siblings etc)
      5. What kind of dwelling do you live in? Ex. House, semi-detached house/townhouse/row house, apartment building, apartment unit within a house, vulnerably housed)
      6. How do you pay for housing? (I.e. own and pay mortgage, rent, live with relative etc.) - is this geared-to-income/subsidized housing?
   7. Are you currently working or volunteering? Is this full or part time?
   8. If you have a spouse at home – do they work? Full or part time?
2. Why did you want to participate in this study when you heard about it?

**Part 2: Experience as a Parent**

1. Tell me a bit about your experience of parenting a young child/young children
2. What are some of the best parts about being a parent?
3. What are some struggles or challenges you face as a parent with young children?
   1. Does anything worry you or keep you up at night?

**Part 3: Financial difficulties**

1. To be eligible for this study, you indicated that you sometimes have trouble making ends meet at the end of the month. Can you tell me a bit more about this?
2. How do you feel about having trouble making ends meet at the end of the month?
   1. How do these feelings impact you?
   2. How do you think it may impact your children?
3. Think back to a time that you were struggling financially and had to go without something you or your child needed. Describe the situation.
   1. How did this make you feel?
   2. What sorts of stresses or hardships did this lead to?
   3. How did you and your family respond to this stress? Probe: Did it cause any conflict? Did it bring you closer together?
   4. How did you resolve the issue?
   5. What did you learn from this experience?
4. Do you feel there are things or experiences that you or your children are missing out on because of your struggles with money? (in other words, your ‘wants,’ not your needs)
5. How do your finances affect your social life? Your children’s social life?

**Use of Community Resources**

1. Have you used any community resources in Kingston to help with things like food, clothing, transportation etc.?
   1. IF no – why is this?
   2. If yes – tell me about your experience accessing these. How did you find out about it/them?
      1. Did you find them helpful?
      2. What are some things you like about resources available in our region?
      3. What do you think is missing? What could be improved?
2. Do you have any final comments about your experience, or is there anything we haven’t covered in our questions that you think is important for us to know?
3. END
